# Effects of Cement Type and Vent Hole Presence on Mass-Based Cement Removal Efficiency and Residual Cement Area in Implant Prostheses

**DOI:** 10.3390/ma19214645

**Published:** 2026-10-31

**Authors:** Ji Un Jang, Kyung Rok Kim, Da Hae Kim, Jung Bo Huh

**Affiliations:** 1 Department of Prosthodontics, School of Dentistry, Pusan National University, Yangsan 50612, Republic of Korea; pnud2020@naver.com (J.U.J.); amotekim@naver.com (K.R.K.); edc205@naver.com (D.H.K.); 2 Dental Studio BN50, Daegu 42785, Republic of Korea; 3 Department of Prosthodontics, Dental Research Institute, Dental and Life Sciences Institute, School of Dentistry, Pusan National University, Yangsan 50612, Republic of Korea

**Keywords:** dental implants, luting cement, residual cement, vent hole, zirconia, implant prosthodontics, cement removal

## Abstract

Residual cement is an important risk factor for peri-implant complications, and both cement properties and prosthetic design may affect its removal. This in vitro study evaluated the effects of four commercial luting cement systems and the presence of a vent hole on mass-based cement removal efficiency and residual cement area around implant abutments. Eighty zirconia restorations were assigned to eight groups (*n* = 10) according to cement type and venting condition. Cement removal efficiency was calculated from mass changes, and residual cement area on the buccal, lingual, mesial, and distal surfaces was quantified using ImageJ. HC3 robust factorial analysis showed significant effects of cement type and venting condition and a significant interaction (*p* = 0.017); after Bonferroni correction, the vented and non-vented conditions differed significantly only for GC FujiCEM II. Linear mixed-effects analysis showed significant effects of cement type, venting condition, and abutment surface, including significant interactions among these factors. Residual cement area was lower in the vented condition for all cements and was generally greater on proximal surfaces. Under the tested in vitro conditions, venting was associated with a higher mass-based index and a lower photographed residual cement area on abutment surfaces, with effects that depended on the cement system and abutment surface. These measurements do not establish improved clinical removal of subgingival excess cement.

## 1. Introduction

Dental implant-supported prosthetic treatment is widely used as a predictable approach for the functional and esthetic rehabilitation of partially and completely edentulous patients [1,2]. The long-term success of implant therapy depends not only on stable osseointegration but also on the mechanical stability of the prosthesis and the maintenance of peri-implant tissue health [3,4]. More recently, treatment goals have expanded beyond implant survival to include the minimization of prosthetic and biological complications and the maintenance of long-term function. From this perspective, the retention type of the implant prosthesis and the cementation procedure are important factors that may influence prosthetic maintenance and peri-implant tissue health [5,6,7,8,9].

Cement-retained implant prostheses eliminate the need for a screw access opening through the restoration, which may interrupt the visible surface or occlusal anatomy, and can facilitate passive fit of the restoration. However, excess cement that is not completely removed during cementation may remain in the peri-implant sulcus around the abutment, and such residual cement has been reported as a risk factor associated with peri-implant mucositis and peri-implantitis [5,6,8,9]. Various approaches have therefore been proposed to reduce these complications, including retrievable screwmentable and screw-retained designs, alternative implant–abutment connections, and techniques that control cement application or margin position [10,11,12,13,14,15]. Nevertheless, cement-retained prostheses may still be selected because of implant position and angulation, esthetic and functional requirements, and available restorative space. Therefore, minimizing cement extrusion and effectively removing residual cement after cementation remain important clinical challenges [6,15].

Various approaches have been investigated to reduce residual cement by modifying the material properties of luting cements and the cementation and cleanup protocols. Cement amount and abutment geometry have been shown to affect cement extrusion [16], and crown-seating methods can influence subgingival cement remnants [17]. The timing and method of excess cement removal may also affect cleanup efficiency. For some cements, a tack-curing protocol involving brief light irradiation before final polymerization has been proposed to facilitate removal of excess cement during the gel phase [18]. However, a study of ceramic crown cementation reported that tack-curing can also affect marginal continuity [19]. Recent in vitro and clinical studies have observed cement-dependent residual cement but have not consistently identified the same cement type as leaving the least residue [20,21]. Studies comparing cement types alongside cementation techniques or cleaning methods indicate that both the material and the applied protocol can affect residual cement [7,20]. Accordingly, differences among commercial cement systems should be interpreted in the context of the cementation and cleanup protocols rather than attributed to a single curing mechanism.

Another strategy is the incorporation of a vent hole in the prosthesis. A vent hole may reduce internal pressure during cementation and provide an escape pathway for cement, thereby affecting the amount of cement extruded at the margin and the pattern of residual cement [22,23,24,25]. Previous studies have reported that the presence, location, and configuration of vent holes can influence cement extrusion and retention. However, it remains unclear whether a standardized vent-hole design produces consistent effects when applied to commercial cement systems with different material properties and cementation/cleanup protocols. Direct comparisons of the same venting design across different cement systems remain limited.

Accordingly, the purpose of this study was to evaluate the effects of different commercial cements and vent hole presence on mass-based cement removal efficiency and residual cement area on implant abutment surfaces in zirconia restorations. Residual cement distribution on the buccal, lingual, mesial, and distal surfaces was also compared, and interactions among cement type, venting condition, and abutment surface were analyzed. The null hypotheses were that (1) cement type and vent hole presence would not affect mass-based cement removal efficiency and there would be no interaction between these factors, and (2) cement type, vent hole presence, and abutment surface would not affect residual cement area and there would be no interactions among these factors.

## 2. Materials and Methods

### 2.1. Study Design and Experimental Groups

**Figure 1:**
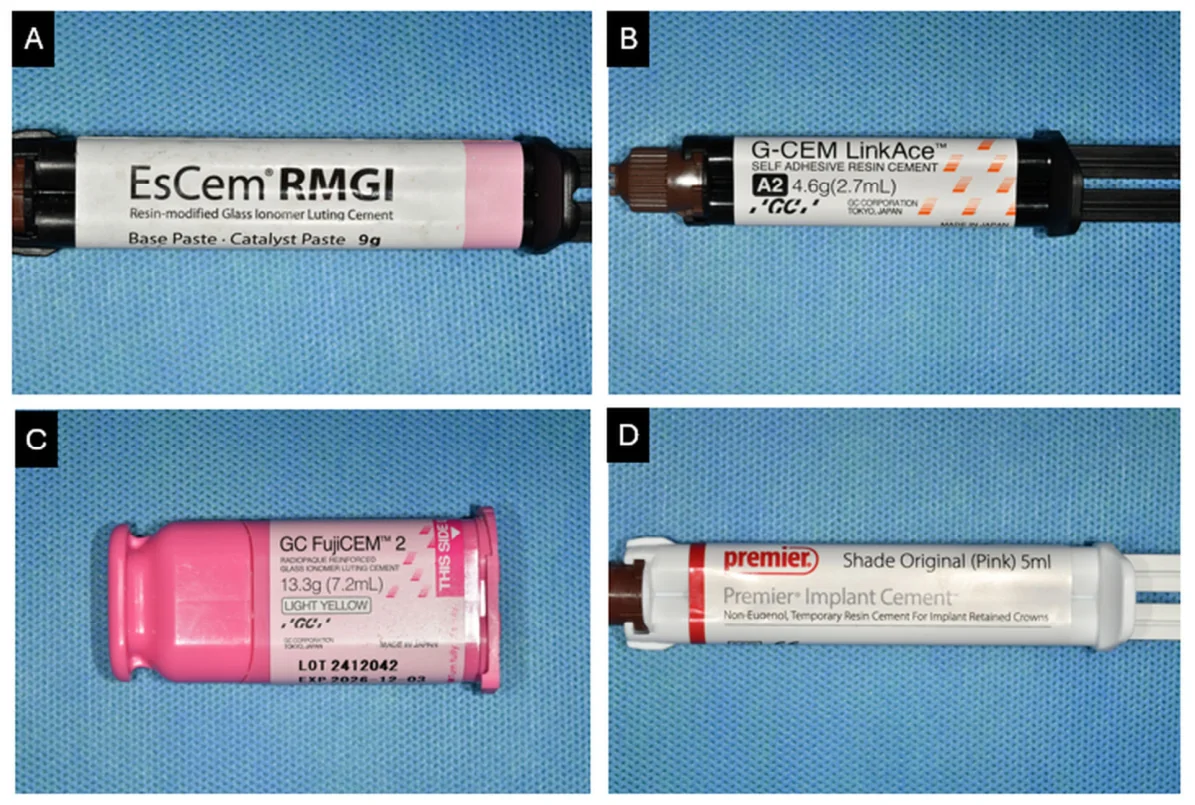
Luting cements used in this study. (**A**) EsCem RMGI; (**B**) G-CEM LinkAce; (**C**) GC FujiCEM II; (**D**) Premier Implant Cement.

Four commercial luting cements used for implant prostheses were evaluated: EsCem RMGI (Spident Co., Ltd., Incheon, Republic of Korea; ER), G-CEM LinkAce (GC Corporation, Tokyo, Japan; GL), GC FujiCEM II (GC Corporation, Tokyo, Japan; FC), and Premier Implant Cement (Premier Dental Products Company, Plymouth Meeting, PA, USA; PI) (Figure 1). The cement type, composition, curing characteristics, and cementation protocol used for each material are summarized in Table 1.

The study used a 4 × 2 factorial design with cement type and vent hole presence as experimental factors. Each cement was tested under vented and non-vented conditions, resulting in eight experimental groups. Ten specimens were assigned to each group, for a total of 80 specimens. No a priori sample size calculation was performed. Specimens were allocated according to a predefined randomization sequence, and the experimental order was also randomized.

Cementation and excess cement removal were performed according to the manufacturers’ instructions and predefined experimental protocols. ER and GL were subjected to tack-curing before excess cement removal, whereas FC and PI were not tack-cured. Therefore, differences among cements were interpreted as differences among complete cementation systems, reflecting both the material properties of each commercial cement and the cementation/cleanup protocol applied to it, rather than as the independent effect of a specific curing mode.

### 2.2. Experimental Model Fabrication

All experiments were performed using a standardized implant model reproducing the mandibular right first molar region (#46). A master model was fabricated from a dentiform (Nissin Dental Products Inc., Kyoto, Japan) using silicone impression material (Imprint II Garant, 3M ESPE, St. Paul, MN, USA). This single posterior model was used to standardize the experimental site; anterior implant sites were not evaluated.

An Osstem TS lab analog (Osstem Implant Co., Seoul, Republic of Korea) was positioned using a custom-made positioning device to maintain an identical location and insertion direction in all experimental models. The analog platform was standardized at 1 mm below the reference alveolar crest. Abutments of identical shape and dimensions were used for all specimens, and a new abutment was used for each specimen without reuse between experiments. The abutment margin was positioned 1.5 mm subgingivally relative to the artificial gingival margin.

**Figure 2:**
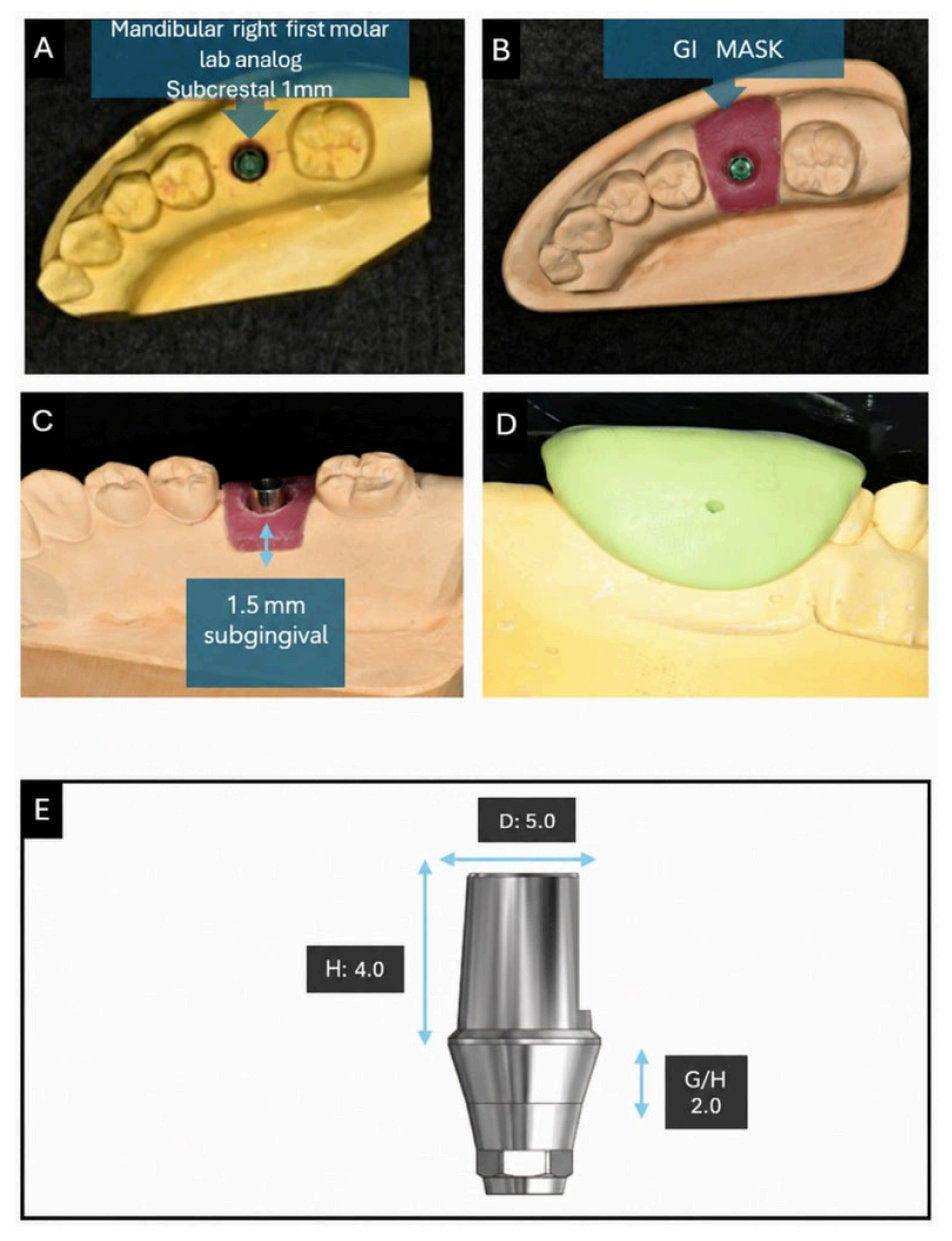
Fabrication of the experimental model. (**A**) Master model. (**B**) Experimental model with artificial gingiva. (**C**) Abutment tightened to the model with a torque of 30 N·cm, with the margin positioned 1.5 mm below the gingival level. (**D**) Silicone putty index for gingival duplication from the master model to the experimental model. (**E**) Abutment design (PNUADD, Busan, Republic of Korea; H = 4.0 mm, G/H = 2 mm, D = 5.0 mm).

Artificial gingiva was fabricated using GI-Mask Automix (Coltene, Altstätten, Switzerland). A silicone putty index (SmartSil Putty; Seil Global Co., Ltd., Busan, Republic of Korea) produced from the master model was used to reproduce the gingival thickness and contour consistently in each experimental model. After fabrication of the artificial gingiva, the vertical distance between the abutment margin and the gingival margin was verified to confirm maintenance of the standardized 1.5 mm subgingival margin (Figure 2).

### 2.3. Fabrication of Zirconia Restorations and Vent Hole Design

**Figure 3:**
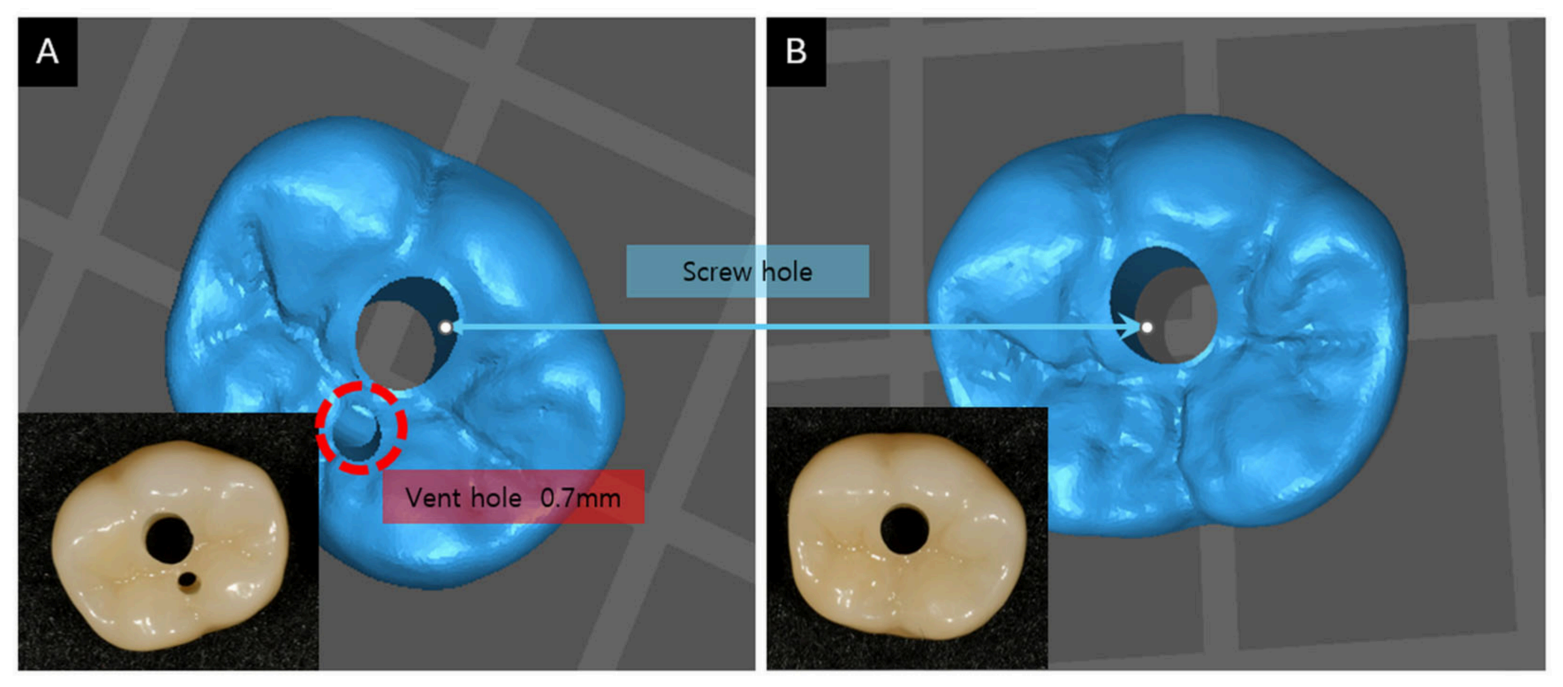
Zirconia restorations with and without a vent hole. (**A**) Vented zirconia restoration with an occlusal screw access hole and an additional 0.7 mm-diameter vent hole on the lingual side. (**B**) Non-vented zirconia restoration with only a central occlusal screw access hole. All restorations were fabricated using a standardized CAD/CAM workflow.

All experimental zirconia restorations were fabricated using an identical CAD design. An occlusal screw-access hole was incorporated to permit access to the implant abutment screw. In the vented groups, an additional 0.7 mm-diameter vent hole was placed on the occlusal surface lingual to the screw-access opening; no additional vent hole was created in the non-vented groups (Figure 3). The 0.7 mm diameter was selected as a small, standardized opening intended to provide an additional cement escape pathway while limiting alteration of the occlusal surface. Patel et al. [24] found that vent-hole diameter did not significantly influence the proportion of cement expressed at the margin across 0.75-, 1.25-, and 1.65 mm openings in an anterior implant-crown model. This finding informed the selection of a small-diameter opening but does not establish that 0.7 mm is optimal or mechanically safe in the present posterior zirconia model. The lingual offset was selected as a design choice to place the opening away from the functional buccal cusp and anticipated occlusal-contact region. Occlusal contacts were not mapped, so this rationale was not mechanically verified. This single configuration was used throughout; lateral vent-hole positions were not evaluated.

The master model was scanned using a FREEDOM HD scanner (DOF, Seoul, Republic of Korea), and the restorations were designed using Ceramill Mind CAD software, version 4.8 (Amann Girrbach, Maeder, Austria). Zirconia restorations were milled from a LUXEN Zr block (E2 shade; Dentalmax Co., Ltd., Cheonan, Republic of Korea) using a Motion 2 CAD/CAM unit (Amann Girrbach, Maeder, Austria). All restorations had identical external and internal designs, and the CAD cement space was set to 50 μm.

After fabrication, complete seating and marginal adaptation were verified for each restoration, and no internal adjustment that could affect fit was performed. Before cementation, the screw access hole was sealed in the same manner for all specimens using a temporary restorative resin (Quick’s Flow Blue, Dentkist, Gunpo, Republic of Korea). This prevented the screw access hole from acting as an unintended cement escape pathway and minimized differences in cement extrusion and retention attributable to pathways other than the experimental vent hole.

### 2.4. Cement Density Measurement and Standardization of Cement Volume

The density of each cement was measured to minimize differences in the applied volume caused by material-specific density. Each cement was mixed according to the manufacturer’s instructions and injected into a 1 cm × 1 cm × 1 cm mold using the same auto-mix-tip filling procedure to minimize air entrapment. The mass of the filled cement was measured using a precision analytical balance (Pioneer series; OHAUS Corporation, Parsippany, NJ, USA). Cement density (ρ) was calculated by dividing the measured mass (m) by the mold volume (V):

ρ = m/V(1)

Density was measured repeatedly under the same conditions, and the mean density was calculated for each cement. The mean densities were 1.8650 g/cm^3^ for ER, 1.7865 g/cm^3^ for GL, 1.8472 g/cm^3^ for FC, and 1.4848 g/cm^3^ for PI (Table 2). These values were used to calculate the target mass corresponding to the same predefined target volume for each cement. During cementation, the converted target mass was used so that the same target cement volume was applied across all groups. The zirconia restoration was weighed immediately before and after cement application to determine the actual cement mass for each specimen, which was then converted to actual applied volume using the measured cement density. This procedure was used to minimize the effects of differences in cement density and application volume on cement extrusion and residual cement formation.

### 2.5. Cementation and Excess Cement Removal

The implant abutments were tightened to each experimental model at 30 N·cm. A cotton pellet of identical size and amount was placed at a standardized depth within the screw access channel. Each cement was mixed according to the manufacturer’s instructions and applied to the internal surface of the zirconia restoration according to the target mass calculated from the measured density so that an equivalent volume was used in all experimental groups. A digital timer was started at the onset of mixing to control the timing of restoration seating, curing, and excess cement removal.

The zirconia restorations were seated along the long axis of the abutment using a custom-made constant-load device. The restorations were advanced at a constant speed of 5 mm/s until an axial load of 50 N was reached, and this 50-N load was maintained during cementation and excess cement removal. The 5 mm/s rate was selected as a previously tested laboratory condition. In a prior in vitro implant-crown study, subgingival remnant cement increased significantly across seating speeds of 5, 10, and 15 mm/s, with the lowest values at 5 mm/s [17]. This finding supports use of 5 mm/s as a controlled experimental rate but does not establish it as an optimal or clinically standard seating speed. A 50-N load was selected as a standardized experimental condition. In a study measuring dentists’ maximum finger pressure during all-ceramic crown cementation, recorded forces ranged from 12 to 67 N [26]; thus, 50 N falls within the observed range, toward its upper end. This finding does not establish that 50 N is routinely maintained throughout clinical cementation or excess cement removal. An implant-crown study also directly compared 25- and 50-N seating forces [17]. Accordingly, the force used here should be interpreted as a controlled laboratory setting rather than an optimal or universally established clinical parameter.

For ER and GL, tack-curing was performed for 5 s and 2 s, respectively, before excess cement removal according to the predefined cementation/cleanup protocols. The same LED light-curing unit (Elipar S10; 3M ESPE, St. Paul, MN, USA) was used, with standardized irradiation distance and direction, and light output was checked before the experiment. Tack-curing was not applied to FC or PI; excess cement removal was initiated at the predetermined curing and cleanup time for each material.

Excess cement was removed by a single operator using the same type of explorer (Hu-Friedy Mfg. Co., Chicago, IL, USA). The cleanup period was limited to 60 s based on preliminary testing, and the start and end times were controlled using a digital timer. The same removal method and sequence were used for all specimens. To minimize temporal bias associated with the starting surface, the initial surface was balanced among the buccal, lingual, mesial, and distal surfaces within each experimental group. All cleanup procedures were performed under identical lighting and magnification conditions.

The artificial gingiva remained in place throughout cementation and excess cement removal. After cleanup was completed, the artificial gingiva was carefully removed to expose the abutment surfaces for subsequent residual cement area analysis. Cement on the artificial gingiva and in the simulated sulcus was not assessed.

### 2.6. Assessment of Mass-Based Cement Removal Efficiency

Mass-based cement removal efficiency was calculated as a quantitative index of excess cement removal. For each specimen, the zirconia restoration was weighed before cement application and again immediately after the predefined amount of cement had been applied to its internal surface. The difference between these two measurements was used to calculate the actual applied cement mass (M_applied).

After completion of excess cement removal, the abutment and zirconia restoration were weighed together. Residual cement mass (M_residual) was defined as the mass measured after cleanup minus the combined pre-cementation mass of the abutment and zirconia restoration. Removed cement mass (M_removed) was calculated as the difference between the applied cement mass and the residual cement mass:

M_removed = M_applied − M_residual(2)

Mass-based cement removal efficiency was calculated as the proportion of removed cement mass relative to the total applied cement mass:

Cement removal efficiency (%) = (M_removed/M_applied) × 100(3)

All mass measurements were performed using the same precision analytical balance. Ambient relative humidity and the weighing environment were not specifically controlled or recorded. In this study, mass-based cement removal efficiency was defined as an experimental index representing the relative proportion of the total applied cement mass that was removed during the excess cement cleanup procedure. Because the denominator also includes cement retained within the restoration as the normal luting layer, this value was not interpreted as equivalent to the clinical removal rate of marginal or subgingival excess cement. The definitions and calculation formulas are summarized in Table 3.

### 2.7. Assessment of Residual Cement Area

After excess cement removal, the artificial gingiva was carefully removed and the buccal, lingual, mesial, and distal surfaces of each abutment were photographed. Specimens were fixed in a custom-made positioning jig designed to reproduce the same position and camera angle, with each evaluated surface positioned perpendicular to the optical axis of the camera.

**Figure 4:**
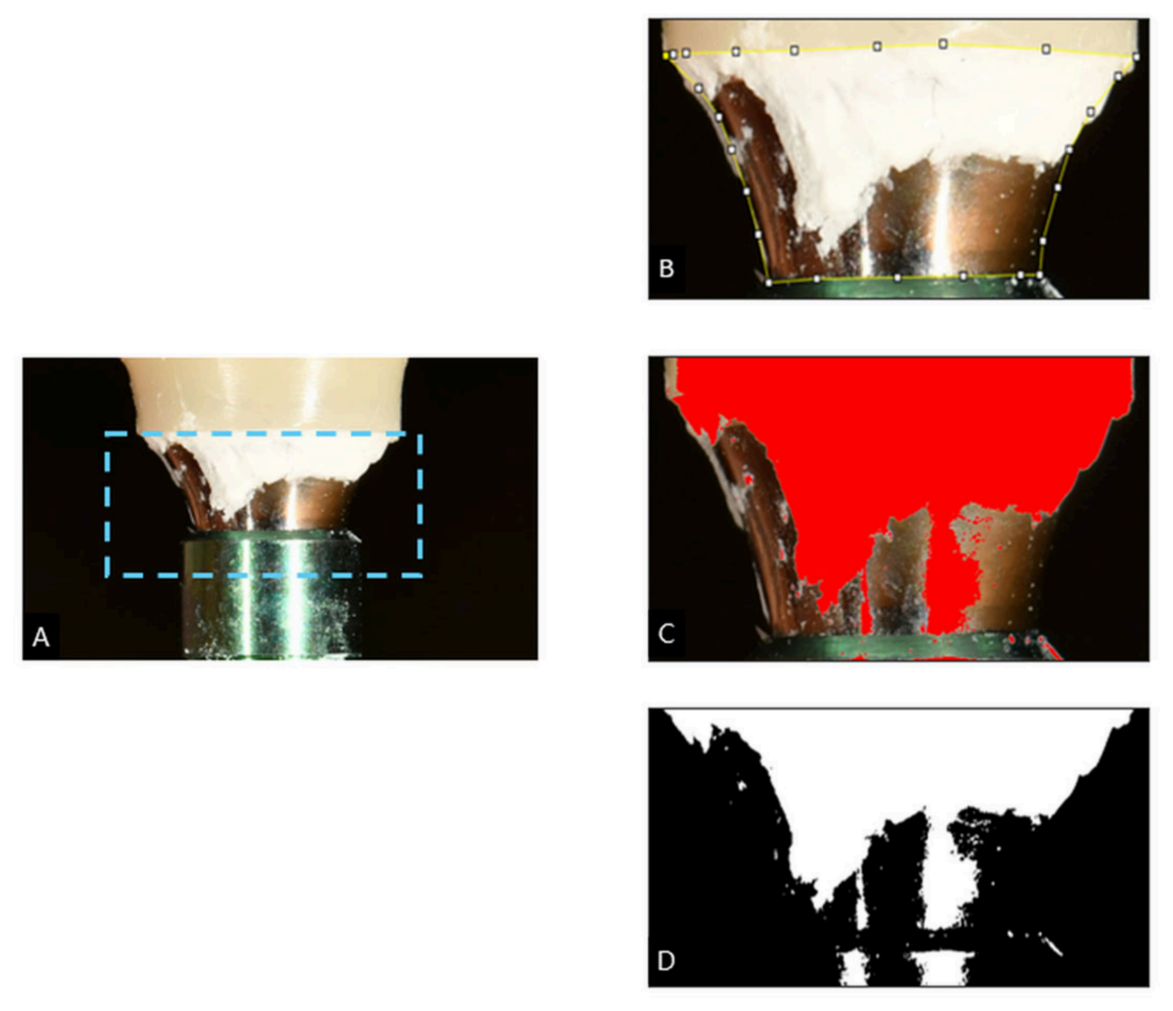
Method for measuring residual cement area. (**A**) Image acquisition at a fixed distance of 0.23 m under standardized lighting conditions (color temperature, 5500 K; ISO 100). (**B**) Selection of the abutment region of interest (ROI). (**C**) Identification of residual cement. (**D**) Binary thresholding and calculation of residual cement area percentage. The dashed cyan rectangle in (**A**) marks the ROI; the yellow boundary and control points in (**B**) show its delineation; the red overlay in (**C**) indicates threshold-identified residual cement, and white pixels in (**D**) represent the segmented area.

Images were acquired using a Nikon D7500 digital camera (Nikon Corp., Tokyo, Japan) equipped with an AF-S VR Micro-Nikkor 105 mm f/2.8 G IF-ED macro lens (Nikon Corp., Tokyo, Japan). The camera-to-specimen distance was fixed at 0.23 m. Lighting was standardized at a color temperature of 5500 K, and ISO 100, magnification, exposure, and white balance were kept constant for all specimens (Figure 4).

Images were analyzed using ImageJ software version 1.54g (National Institutes of Health, Bethesda, MD, USA). A region of interest (ROI) was defined on each image according to the same predefined anatomical criteria, and the same ROI definition was applied to all cement and venting conditions. Images were anonymized using randomized codes so that the analyst was unaware of cement type and venting condition.

A predefined image-processing and thresholding protocol was applied identically to all experimental groups; threshold values were not adjusted according to cement type or venting condition. The segmented residual cement area was divided by the total ROI area to calculate the residual cement area percentage:

Residual cement area (%) = (Residual cement area/ROI area) × 100(4)

Residual cement area was measured separately on the buccal, lingual, mesial, and distal surfaces of each specimen. The four surface-specific measurements obtained from the same specimen were treated as repeated observations in the statistical analysis. The measured percentage represents the two-dimensional projected area covered by cement within each photographed ROI; it does not quantify cement thickness, volume, or mass.

### 2.8. Statistical Analysis

Statistical analyses were performed using IBM SPSS Statistics, version 29.0.2.0 (IBM Corp., Armonk, NY, USA). Mass-based cement removal efficiency was analyzed using a two-way factorial linear model with cement type (ER, GL, FC, and PI) and venting condition (vented and non-vented) as fixed factors. Normality of model residuals was assessed using the Shapiro–Wilk test, and homogeneity of variance was assessed using Levene’s test. When homogeneity of variance was not satisfied, a robust Wald F test using the heteroscedasticity-consistent covariance estimator HC3 was used to evaluate the main effects of cement type and venting condition and their interaction. When a significant interaction was detected, the simple effect of venting within each cement was assessed using HC3 robust contrasts with Bonferroni adjustment for multiple comparisons.

Residual cement area was analyzed using a linear mixed-effects model to account for repeated measurements from the buccal, lingual, mesial, and distal surfaces of the same specimen. Cement type, venting condition, and abutment surface were specified as fixed effects, and specimen was included as a random effect to account for within-specimen correlation. Main effects and interactions among cement type, venting condition, and abutment surface were evaluated. Bonferroni adjustment was applied to post hoc pairwise comparisons for significant main effects or interactions. Statistical significance was set at α = 0.05.

## 3. Results

### 3.1. Mass-Based Cement Removal Efficiency

Normality of model residuals for mass-based cement removal efficiency was not rejected by the Shapiro–Wilk test (W = 0.977, *p* = 0.155), whereas homogeneity of variance was not satisfied according to Levene’s test (F(7,72) = 3.57, *p* = 0.002). Inferential statistics were therefore interpreted using an HC3 heteroscedasticity-consistent robust factorial analysis. Significant main effects were found for cement type (F(3,72) = 53.31, *p* < 0.001) and venting condition (F(1,72) = 26.56, *p* < 0.001), and the interaction between cement type and venting condition was also significant (F(3,72) = 3.62, *p* = 0.017).

Overall, mean cement removal efficiency was 65.3% in the vented condition and 60.4% in the non-vented condition. The mean difference was 5.0 percentage points (95% CI, 3.1–6.9), and the main effect of venting remained significant in the HC3 robust analysis (*p* < 0.001). Because the cement type × venting interaction was significant, however, the magnitude of the venting effect differed among cement systems.

**Figure 5:**
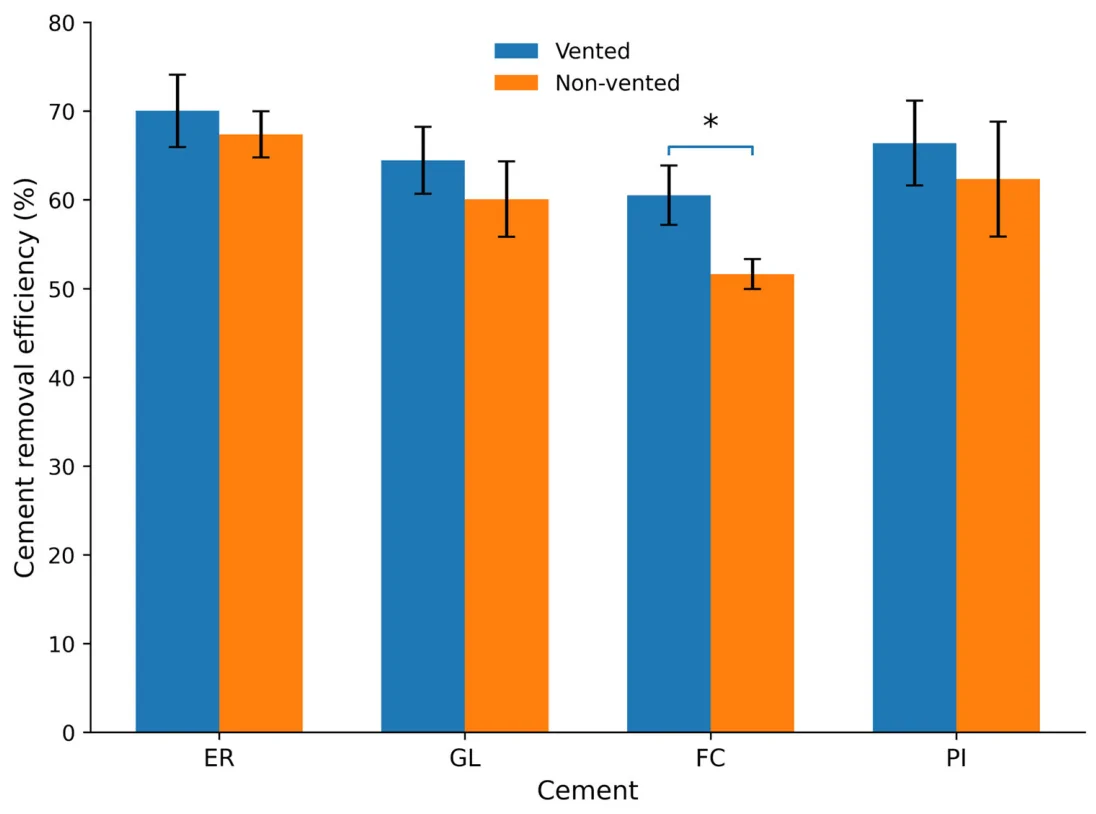
Mass-based cement removal efficiency (%) of four luting cements under vented and non-vented conditions. Bars represent mean values and error bars indicate standard deviations. The asterisk indicates a statistically significant vented–non-vented difference after Bonferroni adjustment (adjusted *p* < 0.001). ER, EsCem RMGI; GL, G-CEM LinkAce; FC, GC FujiCEM II; PI, Premier Implant Cement.

Mean removal efficiency was 67.4 ± 2.6% under the non-vented condition and 70.0 ± 4.1% under the vented condition for ER; 60.1 ± 4.3% and 64.4 ± 3.8%, respectively, for GL; 51.6 ± 1.7% and 60.5 ± 3.3%, respectively, for FC; and 62.3 ± 6.5% and 66.4 ± 4.8%, respectively, for PI (Table 4; Figure 5).

Because of the significant interaction, the simple effect of venting within each cement was analyzed using HC3 robust contrasts with Bonferroni adjustment. The mean vented–non-vented difference was 2.7 percentage points for ER (95% CI, −0.6–5.9; adjusted *p* = 0.421), 4.4 percentage points for GL (95% CI, 0.6–8.2; adjusted *p* = 0.097), 8.9 percentage points for FC (95% CI, 6.4 to 11.4; adjusted *p* < 0.001), and 4.0 percentage points for PI (95% CI, −1.3–9.4; adjusted *p* = 0.545). After correction for multiple comparisons, a significant difference between vented and non-vented conditions was identified only for FC. Thus, mass-based cement removal efficiency was affected by both cement type and venting condition, and the magnitude of the venting effect differed significantly among cement systems, with the largest difference observed for FC.

### 3.2. Residual Cement Area

Linear mixed-effects analysis of residual cement area showed significant main effects of cement type, venting condition, and abutment surface (Wald χ^2^(3) = 1816.63, *p* < 0.001; Wald χ^2^(1) = 271.00, *p* < 0.001; and Wald χ^2^(3) = 1262.39, *p* < 0.001, respectively). Significant two-way interactions were also observed between cement type and venting condition (Wald χ^2^(3) = 21.35, *p* < 0.001), cement type and abutment surface (Wald χ^2^(9) = 97.81, *p* < 0.001), and venting condition and abutment surface (Wald χ^2^(3) = 72.82, *p* < 0.001). The three-way interaction among cement type, venting condition, and abutment surface was also significant (Wald χ^2^(9) = 80.71, *p* < 0.001), indicating that the effect of venting on residual cement area depended on both cement type and the evaluated surface.

Under the vented condition, residual cement areas on the buccal, lingual, mesial, and distal surfaces were 10.1 ± 1.7%, 14.0 ± 2.4%, 18.3 ± 2.5%, and 23.5 ± 2.7%, respectively, for ER; 13.0 ± 1.7%, 14.5 ± 1.6%, 19.1 ± 3.5%, and 23.5 ± 1.5% for GL; 23.5 ± 2.4%, 37.6 ± 3.9%, 38.8 ± 2.7%, and 41.5 ± 3.8% for FC; and 16.1 ± 2.0%, 22.4 ± 2.5%, 25.9 ± 2.1%, and 31.0 ± 2.9% for PI (Table 5).

Under the non-vented condition, residual cement areas on the buccal, lingual, mesial, and distal surfaces were 16.8 ± 3.0%, 17.6 ± 2.4%, 23.0 ± 2.7%, and 27.2 ± 2.9%, respectively, for ER; 18.4 ± 2.7%, 18.5 ± 1.8%, 25.5 ± 2.8%, and 26.7 ± 1.8% for GL; 42.1 ± 3.8%, 43.6 ± 3.9%, 45.2 ± 3.7%, and 46.0 ± 3.3% for FC; and 24.9 ± 1.9%, 27.3 ± 1.6%, 34.8 ± 1.8%, and 38.7 ± 4.3% for PI (Table 6).

When the four surfaces of each specimen were averaged, mean residual cement area under vented versus non-vented conditions was 16.5 ± 1.1% versus 21.2 ± 2.0% for ER, 17.5 ± 1.2% versus 22.3 ± 1.9% for GL, 35.4 ± 1.3% versus 44.2 ± 3.3% for FC, and 23.9 ± 1.3% versus 31.4 ± 1.7% for PI. For all four cements, mean residual cement area was significantly lower in the vented condition than in the non-vented condition (Bonferroni-adjusted *p* < 0.001).

The mean reduction associated with venting was 4.7 percentage points for ER (95% CI, 3.1–6.2), 4.8 percentage points for GL (95% CI, 3.2–6.3), 8.9 percentage points for FC (95% CI, 7.3–10.4), and 7.6 percentage points for PI (95% CI, 6.0–9.1). Thus, the reduction in residual cement area associated with venting was relatively greater for FC and PI than for ER and GL.

Surface-specific post hoc analysis showed that in the vented ER group residual cement area increased significantly in the order buccal < lingual < mesial < distal. In the vented GL group, no significant difference was observed between the buccal and lingual surfaces, whereas the mesial and distal surfaces showed significantly greater residual cement area. In the vented FC group, the buccal surface showed significantly less residual cement than the other surfaces; no significant difference was observed between the lingual and mesial surfaces, and the distal surface showed the highest value. In the vented PI group, residual cement area increased significantly in the order buccal < lingual < mesial < distal.

In the non-vented ER group, no significant difference was observed between the buccal and lingual surfaces, whereas residual cement area increased significantly on the mesial and distal surfaces. In the GL group, no significant difference was observed between the buccal and lingual surfaces or between the mesial and distal surfaces; however, the proximal surfaces showed greater residual cement area than the buccal and lingual surfaces. In the FC group, the buccal surface showed significantly less residual cement than the mesial and distal surfaces, whereas differences among the remaining surfaces were limited. In the PI group, no significant difference was observed between the buccal and lingual surfaces, whereas the mesial and distal surfaces showed significantly greater residual cement area.

Overall, mean residual cement area was 23.3% under the vented condition and 29.8% under the non-vented condition. Across most experimental groups, residual cement area tended to be greater on the mesial and distal surfaces than on the buccal and lingual surfaces.

## 4. Discussion

This study evaluated the effects of four commercial cement systems and vent hole presence on mass-based cement removal efficiency and residual cement area on implant abutment surfaces in zirconia restorations. Residual cement distribution was also compared across the buccal, lingual, mesial, and distal surfaces, and interactions among cement type, venting condition, and abutment surface were assessed. In the HC3 robust analysis of mass-based cement removal efficiency, both main effects and the cement type × venting interaction were significant. For residual cement area, the main effects of cement type, venting condition, and abutment surface, as well as their interactions, were significant. Accordingly, both null hypotheses were rejected. These findings indicate that cement removal and retention after cementation of implant prostheses may be jointly influenced by cement material properties, the cementation and cleanup protocol, venting design, and the abutment surface on which residual cement is located.

### 4.1. Cement Type and Cementation/Cleanup Protocol

Selection of a luting cement for implant prostheses requires consideration not only of retention but also of retrievability and the ease of excess cement removal. Kim et al. [27] reported that retention characteristics differ among cements used for implant-supported restorations. In the present study, cement type significantly affected both mass-based cement removal efficiency and residual cement area. Overall, removal efficiency was highest for ER and lowest for FC, while residual cement area also differed markedly among cement systems. These differences may reflect the combined effects of cement composition, viscosity, flowability, and polymerization behavior together with differences in the applied cementation and cleanup protocols. Çıklaçifci and Kara [20] reported that cleaning with a PEEK ultrasonic tip reduced residual cement compared with an explorer alone, regardless of cement type. Among the tested materials, resin cement left the least residue, whereas glass ionomer and polycarboxylate cements left more. Conversely, Weinreuter et al. [21] found larger residual cement-covered areas with resin composite cement than with conventional glass ionomer cement in a randomized clinical trial. These differing findings do not establish a universal ranking of cement classes. In the present study, ER and FC, both classified as resin-modified glass ionomer cements, also showed different residual cement areas. Differences in commercial formulations, removal protocols, and experimental or clinical conditions may contribute to the differing patterns, but the present design cannot identify their independent contributions.

ER and GL were used with protocols that included tack-curing before excess cement removal. Partial light polymerization through tack-curing has been reported to alter cement properties before final polymerization and facilitate removal of excess cement during the gel phase [18]. Evidence from ceramic crown cementation also indicates that tack-curing can affect marginal continuity [19]. However, the present study was not designed to compare tack-curing and non-tack-curing independently within the same cement. Therefore, the differences observed among cement groups cannot be attributed specifically to tack-curing itself. Rather, the findings should be interpreted as outcomes of the complete cementation system, including the material properties of each commercial cement and the cementation/cleanup protocol applied to it.

### 4.2. Effect of the Vent Hole

A vent hole may reduce pressure generated within the restoration during cementation and provide a cement escape pathway, thereby influencing cement extrusion and retention at the restoration margin. Previous studies have similarly reported that the presence, position, and configuration of a vent hole can affect cement extrusion and residual cement formation [22,23,24,25]. Ye et al. [25] separately measured cement extruded through vent holes and at the cervical margin. They found less cervical cement extrusion with an occlusal vent hole than without a vent hole, and extrusion varied with vent location. The lower photographed residual cement area in the vented groups of the present study is compatible with altered cement escape pathways. However, marginal extrusion before cleanup and residual surface coverage after cleanup are different outcomes; the present findings do not establish whether venting improved removal of cement already present at the margin. Ye et al. [25] also compared occlusal and lateral vent holes in posterior implant crowns. In their model, both positions reduced cervical cement extrusion relative to no vent hole, while the occlusal-hole group showed less cervical extrusion than the lateral-hole groups. They also described potential esthetic drawbacks of occlusal openings, indicating that vent location may involve trade-offs. Because the present study tested only one 0.7 mm occlusal/lingual configuration, it cannot determine whether lateral placement would produce similar cement escape or residual-cement patterns.

Because homogeneity of variance was not satisfied for mass-based cement removal efficiency, an HC3 robust factorial analysis was used. Venting had a significant main effect (*p* < 0.001), and the interaction between cement type and venting condition was also significant (*p* = 0.017). Thus, although removal efficiency was generally higher under vented conditions, the magnitude of the increase differed among cement systems. Bonferroni-adjusted simple-effect analysis identified a significant vented–non-vented difference only for FC (adjusted *p* < 0.001), whereas ER, GL, and PI did not show significant within-cement differences. Therefore, the effect of a vent hole on mass-based cement removal efficiency should not be generalized as having the same magnitude across all cements; rather, its effect appears to depend on the cement system.

Residual cement area showed a somewhat different pattern. The linear mixed-effects model demonstrated not only a significant main effect of venting but also significant cement type × venting and cement type × venting × abutment surface interactions. When the four surfaces were averaged, residual cement area was lower under the vented condition for all four cements, and the mean reduction associated with venting was relatively greater for FC and PI than for ER and GL. However, because the three-way interaction was significant, the reduction in residual cement associated with venting should not be explained solely by cement type; the abutment surface on which residual cement is located must also be considered.

The two outcome measures therefore describe different aspects of cement distribution after cementation and cleanup. Ye et al. [25] distinguished cement exiting the vent from cement exiting the cervical margin, whereas the present mass-based index does not distinguish these pathways or separate the internal luting layer from marginal residues. Consequently, the mass-based index should not be directly equated with their marginal extrusion measurements or with the efficiency of subgingival excess cement removal. The photographic outcome provides location-specific surface coverage but does not resolve this mass distribution.

### 4.3. Surface-Specific Distribution of Residual Cement

The location of residual cement is clinically important in addition to its total amount. Subgingival residual cement can be difficult to identify and remove visually, and the probability of residual cement has been reported to increase as the abutment margin is positioned more deeply subgingivally [28].

In this study, the buccal, lingual, mesial, and distal surfaces of each abutment were evaluated separately, and abutment surface had a significant effect on residual cement area. Overall, greater residual cement area tended to occur on the mesial and distal surfaces than on the buccal and lingual surfaces. This finding may reflect the anatomical form of proximal surfaces and the limitations in visibility and instrument access during excess cement removal.

However, significant interactions were identified between cement type and abutment surface, between venting condition and abutment surface, and among cement type, venting condition, and abutment surface. Therefore, it cannot be generalized that an identical degree of residual cement accumulation occurs on proximal surfaces for all cements and under all conditions. In some groups, differences between the buccal and lingual surfaces or between the mesial and distal surfaces were limited, and surface-specific residual patterns varied according to cement and venting condition. Clinically, careful inspection and cleanup of proximal surfaces remain particularly important, but the location of residual cement should not be interpreted as a simple surface effect independent of cement system and prosthetic design. Weinreuter et al. [21] found no significant differences in residual cement ratios among mesial, distal, buccal, and oral surfaces in their clinical trial, in contrast to the surface-dependent pattern observed here. Their clinical setting, individualized margin positions, cement products, and cementation conditions differed from the standardized artificial-gingiva model used in the present study. These differences may contribute to the contrasting observations, although they cannot be isolated by cross-study comparison. Thus, the proximal predominance observed here should be confined to the tested conditions rather than presented as a universal clinical distribution.

### 4.4. Interpretation of Residual Cement Area and Mass-Based Cement Removal Efficiency

Residual cement area on the abutment surface and mass-based cement removal efficiency were used as complementary outcome measures. Image-based residual cement area provides a direct assessment of the distribution and relative surface area of cement remaining on the abutment after cleanup. In contrast, mass-based cement removal efficiency quantifies the proportion of the total applied cement mass that is removed during cleanup by using the mass differences before cementation, after cement application, and after excess cement removal.

The denominator of mass-based cement removal efficiency includes cement that remains within the restoration as the normal luting layer after cementation. Therefore, this value should not be interpreted as equivalent to the clinical removal rate of excess cement that should be eliminated from the marginal or subgingival area. Moreover, the mass-based outcome does not provide information on the location of the remaining cement. The different interaction patterns observed for mass-based cement removal efficiency and residual cement area may reflect the fact that these variables represent different aspects of cement cleanup. Accordingly, mass-based removal efficiency should be regarded as a complementary quantitative index and interpreted together with direct assessment of residual cement area on the abutment surface.

### 4.5. Significance of Experimental Standardization

Cement extrusion and residual cement may be influenced by cement amount, abutment geometry, and restoration-seating methods [16,17]. To minimize the effects of these variables, cement density was measured so that an equivalent cement volume could be applied, and a constant-load device was used to standardize seating force and seating speed. The timing of cement removal and the cleanup duration were predefined, and the starting surface was balanced among experimental groups to minimize temporal bias related to the cleanup sequence. Ji et al. [17] reported increasing subgingival remnant cement with increasing seating speed, seating force, and applied cement quantity, with material-dependent differences. Their findings support controlling these procedural variables when comparing cement systems. However, their assessment of subgingival cement by three-dimensional scanning and mass measurement differs from the present photographic surface analysis and total-applied-mass index, so numerical outcomes are not directly comparable. In that study, the 50-N group had greater subgingival remnant cement than the 25-N group [17]. Because the present experiment used a single seating force, the independent effect of force could not be assessed, and the selected load may have influenced cement extrusion and residual cement formation.

For image analysis of residual cement, camera distance, lighting conditions, acquisition settings, ROI definition, and segmentation protocol were standardized. Images were anonymized so that cement type and venting condition were concealed during analysis. These procedures were intended to reduce the likelihood that variation related to the operator, cementation procedure, or measurement process would be misinterpreted as an effect of cement type or vent hole presence.

### 4.6. Limitations

Several limitations should be considered. First, this was an in vitro study and could not fully reproduce the biological characteristics of peri-implant soft tissues or the clinical environment. Although artificial gingiva was used to standardize the subgingival environment, the elasticity of natural soft tissue, the presence of fluids, tissue pressure, and patient-specific anatomical variation were not reproduced. Consequently, accessibility during cleanup and residual cement distribution in this model may differ from those in clinical peri-implant tissues.

Second, the experiment was performed under standardized conditions using a mandibular first molar site, a single abutment geometry, a 1.5 mm subgingival margin, a specific 0.7 mm vent hole diameter and location, and a 50-μm CAD cement space. Therefore, the findings cannot be directly generalized to other implant positions, abutment geometries, margin depths, cement spaces, or vent hole positions and dimensions. The posterior #46 model does not represent anterior implant restorations, where esthetic demands and margin placement may differ; the findings should not be extrapolated to anterior sites. In addition, the zirconia region between the screw-access opening and vent hole was not mechanically evaluated, so its fracture resistance and fatigue behavior remain unknown. Occlusal contacts were not mapped; therefore, the extent to which the lingual offset reduced direct contact loading was not verified.

Third, this study compared four commercial cements together with the cementation and cleanup protocol applied to each. It was not designed to isolate the independent effects of individual chemical or rheological properties or of tack-curing itself. Because cement-specific curing and cleanup protocols were combined with a fixed 60 s cleanup period, between-group differences cannot be attributed independently to cement composition, viscosity, flowability, or curing behavior. Additional studies in which curing and cleanup protocols are varied within the same cement are required to clarify these individual effects.

Fourth, mass-based cement removal efficiency was calculated relative to the total applied cement, including the normal luting layer remaining inside the restoration, and therefore does not directly represent the clinical removal rate of subgingival excess cement. For this reason, it was interpreted together with residual cement area on the abutment surface. Ambient relative humidity and the weighing environment were not standardized, and the exact interval preceding each weighing was not recorded separately. Therefore, potential time-dependent mass changes due to water loss or moisture exchange could not be quantified and may have influenced the gravimetric measurements.

Fifth, photographic analysis measures two-dimensional projected surface coverage and cannot quantify residual cement thickness, volume, or mass. Standardized imaging does not eliminate projection distortion or overlap on curved surfaces, and surface coverage alone does not establish the clinical significance of the remaining cement. Sixth, only one type of explorer was used for excess cement removal. Different removal instruments or cleanup techniques may produce different results. Seventh, no a priori sample size calculation was performed. With 10 specimens per group, the study may have had limited power to detect small effects, particularly interaction effects; therefore, nonsignificant findings should be interpreted cautiously. Finally, cement on the artificial gingiva or in the simulated sulcus was not quantified; this omission may underestimate total peri-implant residue and overestimate effective clearance. Differences in the optical properties of the cement materials may also have affected threshold-based segmentation; this possibility was not independently validated. Formal intra- or inter-observer repeatability and segmentation accuracy were not assessed, and no three-dimensional imaging or measurement was performed. Accordingly, the residual-area results should be interpreted as standardized two-dimensional surface-coverage measurements rather than validated estimates of cement thickness, volume, or mass.

Taken together, the findings indicate that minimizing residual cement after cementation of implant prostheses requires consideration of the cement system, cementation and cleanup protocol, venting design, and the abutment surface on which residual cement may remain, rather than focusing on a single material property or on the presence of a vent hole alone. Venting generally increased mass-based cement removal efficiency and reduced residual cement area, but the magnitude of these effects differed according to cement type and, for residual cement area, according to abutment surface. Because this study was conducted under standardized in vitro conditions, additional investigations under different prosthetic and clinical conditions are required before specific clinical recommendations can be made.

## 5. Conclusions

Within the limitations of this in vitro study, cement type and vent hole presence affected the mass-based cement removal index calculated from the total applied cement mass, with a significant interaction between these factors. The index was generally higher under vented conditions, but the magnitude of the difference depended on the cement system; after Bonferroni adjustment, a significant vented–non-vented difference was identified only for FC. Because this index includes cement retained within the restoration as the normal luting layer and does not isolate subgingival excess cement, these findings do not establish that venting improves clinical removal of subgingival cement.

Photographed residual cement area on the abutment surfaces was generally lower under the vented condition, and the effect varied according to cement type and abutment surface. Photographed residual area also tended to be greater on the mesial and distal surfaces than on the buccal and lingual surfaces.

These results describe changes in the mass-based index and photographed abutment-surface coverage under the tested in vitro conditions; clinical benefit cannot be inferred. Further studies under a broader range of prosthetic and clinical conditions are required to determine the clinical implications of these findings.

## Figures and Tables

**Table 1:** Luting cements used in this study.

Groups	Cement	Type	Powder Components	Liquid Components/ Resin Matrix	Curing Protocol	Venting/*n*
ER-V/ ER-NV	EsCem RMGI (ER)	RMGI	Fluoroaluminosilicate glass; resin-modified fillers; radiopaque fillers	HEMA; polycarboxylic acid copolymer; water; camphorquinone–amine photoinitiator	Tack-curing, 5 s	Vented/non-vented; *n* = 10 each
GL-V/ GL-NV	G-CEM LinkAce (GL)	Resin cement	Silica fillers; fluoroaluminosilicate glass; radiopaque glass fillers	UDMA; MDP; dimethacrylates; CQ–amine initiator system; additives/stabilizers	Tack-curing, 2 s	Vented/non-vented; *n* = 10 each
FC-V/ FC-NV	GC FujiCEM II (FC)	RMGI	Fluoroaluminosilicate glass; radiopaque fillers	Polyacrylic acid; HEMA; photoinitiator (when light-activated)	No tack-curing	Vented/non-vented; *n* = 10 each
PI-V/ PI-NV	Premier Implant Cement (PI)	Resin cement	Radiopaque glass fillers; silica-based fillers	Bis-GMA; TEGDMA; UDMA; CQ-based photoinitiator; additives/stabilizers	No tack-curing	Vented/non-vented; *n* = 10 each

**Table 2:** Density of each luting cement.

Cement	Density (g/cm^3^)
EsCem RMGI (ER)	1.8650
G-CEM LinkAce (GL)	1.7865
GC FujiCEM II (FC)	1.8472
Premier Implant Cement (PI)	1.4848

**Table 3:** Definitions and formula used to calculate mass-based cement removal efficiency.

Parameter	Definition/Formula
A	Abutment + zirconia restoration before cementation
B	Abutment + zirconia restoration after cement application
C	Abutment + zirconia restoration after removal of excess cement
Applied cement	B − A
Residual cement	C − A
Removed cement	B − C
Removal efficiency (%)	(B − C)/(B − A) × 100

**Table 4:** Mass-based cement removal efficiency (%) according to venting condition and cement type.

Cement	Vented (Mean ± SD)	Non-Vented (Mean ± SD)
EsCem RMGI (ER)	70.0 ± 4.1	67.4 ± 2.6
G-CEM LinkAce (GL)	64.4 ± 3.8	60.1 ± 4.3
GC FujiCEM II (FC)	60.5 ± 3.3	51.6 ± 1.7
Premier Implant Cement (PI)	66.4 ± 4.8	62.3 ± 6.5

HC3 robust factorial analysis showed significant effects of cement type (*p* < 0.001), venting condition (*p* < 0.001), and their interaction (*p* = 0.017). Bonferroni-adjusted simple-effect analysis showed a significant vented–non-vented difference only for FC (adjusted *p* < 0.001).

**Table 5:** Residual cement area (%) by abutment surface and cement type in the vented groups.

Cement	Buccal	Lingual	Mesial	Distal
EsCem RMGI (ER)	10.1 ± 1.7	14.0 ± 2.4	18.3 ± 2.5	23.5 ± 2.7
G-CEM LinkAce (GL)	13.0 ± 1.7	14.5 ± 1.6	19.1 ± 3.5	23.5 ± 1.5
GC FujiCEM II (FC)	23.5 ± 2.4	37.6 ± 3.9	38.8 ± 2.7	41.5 ± 3.8
Premier Implant Cement (PI)	16.1 ± 2.0	22.4 ± 2.5	25.9 ± 2.1	31.0 ± 2.9

Values are presented as mean ± SD. Residual cement area was analyzed using a linear mixed-effects model with cement type, venting condition, and abutment surface as fixed effects and specimen as a random effect. Pairwise comparisons were adjusted using the Bonferroni method.

**Table 6:** Residual cement area (%) by abutment surface and cement type in the non-vented groups.

Cement	Buccal	Lingual	Mesial	Distal
EsCem RMGI (ER)	16.8 ± 3.0	17.6 ± 2.4	23.0 ± 2.7	27.2 ± 2.9
G-CEM LinkAce (GL)	18.4 ± 2.7	18.5 ± 1.8	25.5 ± 2.8	26.7 ± 1.8
GC FujiCEM II (FC)	42.1 ± 3.8	43.6 ± 3.9	45.2 ± 3.7	46.0 ± 3.3
Premier Implant Cement (PI)	24.9 ± 1.9	27.3 ± 1.6	34.8 ± 1.8	38.7 ± 4.3

Values are presented as mean ± SD. Residual cement area was analyzed using a linear mixed-effects model with cement type, venting condition, and abutment surface as fixed effects and specimen as a random effect. Pairwise comparisons were adjusted using the Bonferroni method.

## Data Availability

The original contributions presented in this study are included in the article. Further inquiries can be directed to the corresponding author.
